# A Description of *Biremis panamae* sp. nov., a New Diatom Species from the Marine Littoral, with an Account of the Phylogenetic Position of *Biremis* D.G. Mann et E.J. Cox (Bacillariophyceae)

**DOI:** 10.1371/journal.pone.0114508

**Published:** 2014-12-10

**Authors:** Andrzej Witkowski, Frederik Barka, David G. Mann, Chunlian Li, Jascha L. F. Weisenborn, Matt P. Ashworth, Krzysztof J. Kurzydłowski, Izabela Zgłobicka, Sławomir Dobosz

**Affiliations:** 1 Palaeoceanology Unit, Faculty of Geosciences, University of Szczecin, Mickiewicza 18, PL-70-383, Szczecin, Poland; 2 Institute of Molecular Biosciences, Goethe University, D-60438 Frankfurt am Main, Germany; 3 Royal Botanic Garden Edinburgh, 20A Inverleith Row, Edinburgh EH3 5LR, Scotland, United Kingdom; 4 Aquatic Ecosystems, Institute for Food and Agricultural Research and Technology (IRTA), Crta de Poble Nou Km 5.5, E-43540 Sant Carles de la Ràpita, Catalunya, Spain; 5 Institute of Ecology, Evolution and Diversity, Goethe University, D-60438 Frankfurt am Main, Germany; 6 Section of Integrative Biology, Biological Laboratories, University of Texas at Austin, Austin, Texas, United States of America; 7 Faculty of Engineering and Material Science, Warsaw University of Technology, Warsaw, Poland; The Evergreen State College, United States of America

## Abstract

Here we present a formal description of *Biremis panamae* Barka, Witkowski et Weisenborn sp. nov., which was isolated from the marine littoral environment of the Pacific Ocean coast of Panama. The description is based on morphology (light and electron microscopy) and the *rbc*L, *psb*C and SSU sequences of one clone of this species. The new species is included in *Biremis* due to its morphological features; i.e. two marginal rows of foramina, chambered striae, and girdle composed of numerous punctate copulae. The new species also possesses a striated valve face which is not seen in most known representatives of marine littoral *Biremis* species. In this study we also present the relationship of *Biremis* to other taxa using morphology, DNA sequence data and observations of auxosporulation. Our results based on these three sources point to an evolutionary relationship between *Biremis*, *Neidium* and *Scoliopleura*. The unusual silicified incunabular caps present in them are known otherwise only in *Muelleria*, which is probably related to the Neidiaceae and Scoliotropidaceae. We also discuss the relationship between *Biremis* and the recently described *Labellicula* and *Olifantiella*.

## Introduction

The diatom genus *Biremis* was established by D.G. Mann et E.J. Cox in Round et al. 1990 ([1], [2]). Included in this new genus were taxa which originally had been described as representatives of several genera, e.g. *Pinnularia*, *Navicula* and *Amphora* (e.g. [3], [4], [5], [6], [7]). *Pinnularia ambigua* Cleve  =  *Biremis ambigua* (Cleve) D.G. Mann was chosen as type of the genus and species that conform to its morphological characteristics were included in *Biremis*. Most taxa belonging to *Biremis* in general inhabit the marine littoral zone; however, some freshwater taxa included in *Oestrupia*, have also been transferred to *Biremis* e.g. *B*. *zachariasii* (Reichelt) Edlund, Andresen & Soninkhishig and *B. undulata* (Schulz) Andresen, Edlund & Soninkhishig ([8]), and Vyverman et al. 1997 ([9]) have described a few *Biremis* species from freshwater habitats in Tasmania. Some *Biremis* taxa, including *B*. *ambigua* (Cleve) D.G. Mann and *B*. *lucens* (Hustedt) Sabbe, Witkowski et Vyverman, seem to have a worldwide distribution (e.g. [2], [10], [11]). However, in general *Biremis* species are poorly known in terms of their biogeography. The total number of *Biremis* species recognized is currently ca. 17, ([1], [12], [10], [11], [13]), with a further two species sometimes separated into the genus *Pulchella* Krammer (see Discussion).

The original separation of *Biremis* from other naviculoid genera was based on its frustule and chloroplast morphology. The following criteria were regarded as of primary significance for *Biremis* by Round et al. 1990 ([1]) and ([2]):

- the external appearance of the striae, containing large unoccluded rounded foramina arranged in two rows, one on the valve face and the other on the valve mantle – hence the genus name *Biremis*, referring to the fast warships of ancient Greece and Rome with their two ranks of oars.
- a valve interior with transapically-elongate chambers with finely porous, sieve-like internal walls, similar to those present in *Scoliopleura* Grunow, *Scoliotropis* Cleve and *Progonoia* Schrader (cf. [14], [1]). This feature caused the genus to be included in the family Scoliotropidaceae as circumscribed by Round et al. 1990 ([1]).
- a girdle composed of several open copulae bearing one or a few rows of poroids.
- usually two chloroplasts per cell, disposed one towards each pole as in *Nitzschia* and some *Amphora* (e.g. *A. ostrearia* Brébisson: [15], rather than the side-by-side arrangement that is more common in naviculoid diatoms. Each chloroplast consists of two large plates connected by a narrow bridge containing a single compact, ± isodiametric pyrenoid.

However, the morphology of taxa included in *Biremis* shows some variability in published sources. So far, three types of morphology can be distinguished. Type 1 has been found only in freshwater forms. Here the external foramina on the valve face are elongate and slit-like – either straight (e.g. *B. hartzii* W. Vyverman, Sabbe et R. Vyverman), curved (e.g. *B. leeawuliana* W. Vyverman, Sabbe et R. Vyverman) or wavy (*B. clarensis* W. Vyverman, Sabbe et R. Vyverman) – rather than round or elliptical. The valve face is free of areolae, apart from the foramina, so that in the light microscope (LM) valves appear to have a wide plain axial area. The second and third types of morphology are represented in marine species. Type 2 morphology conforms to the generic type and characterizes most of the *Biremis* species that have been described thus far. In it, the valve possesses the usual two rows of rounded foramina externally, with one of them located along the margin of the valve face and the second one along the valve mantle, and the valve face is free of areolae (see [1], [2], [10]), creating a wide plain axial area as in type 1. Type 3 morphology has been observed in a few taxa, of which only one has been formally described, i.e. *B*. *solitaria* (Cleve) Witkowski, Lange-Bertalot & Metzeltin ( =  *Navicula solitaria* of Cleve 1896 [16]), and two are unnamed, one described from the Gulf of Gdańsk ([11]) and the other illustrated from North Wales ([2], Fig. 3: 22, 24, 26). In these species, in addition to the two rows of foramina located along the margin of the valve face and along the mantle, the valves possess areolae on the valve face.

*Biremis panamae*, described for the first time in this paper from the tropical Pacific Ocean (coast of Panama), possesses this third type of morphology, whereas a second species, whose *rbc*L was sequenced earlier for us by G.R. Simpson, M. Hollingsworth and A. Clark (results included here), possesses type 2 morphology. These two species therefore provide an excellent opportunity to use morphological and molecular data to establish the phylogenetic position of *Biremis*. The aim of this paper is therefore to formally describe *B. panamae* and use morphological, gene sequence, and auxosporulation data to explore the relationship between *Biremis* and other raphid diatoms.

## Materials and Methods

### Isolation, culturing and microscopy of vegetative material

**Figure 1:**
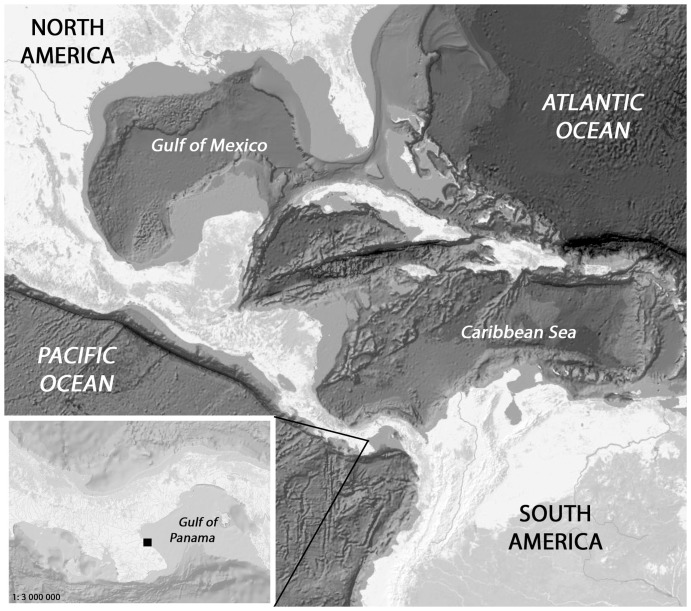
Location of the sampling site on the coast of Panama.

A clonal culture was isolated from specimens collected on January 20^th^ 2011 along the drift line in the Pacific Ocean on the public beach "Playa Monagre" in Los Santos Province, Panama (7° 58′ 32.46″ S and 80° 20′ 50.31″ W, **Fig. 1**). No specific permissions were required for this location and sampling activity. The field studies did not involve endangered or protected species. The Gulf of Panama, where the sample was collected, is considered a large semi-open upwelling area. The average surface water temperature is about 26°C but temperatures, as well as the distributions of nutrients in the Gulf, change due to seasonal wind-driven upwelling events. Thus, during the dry season, water temperatures can decrease to 16°C while nutrients, e.g. NO_3_^−^ and PO_4_^3−^ can reach relatively high concentrations compared to the rainy season ([17]).

Isolates were inoculated into enriched seawater (f/2)-medium [18] and maintained at 18°C under a 16 h light − 8 h dark cycle, illuminated by 50 µmol m^−2^ s^−1^ of white light for about six weeks. After the cultures became visibly coloured, algal cell counts were determined using a Thoma counting chamber (Hecht–Assistent, Sondheim, Germany) and approximately 250 algal cells were streaked out on f/2-agar plates containing 1.5% (w/v) agar (AppliChem, Darmstadt, Germany). After 2–3 weeks of further incubation at the described culture conditions, single cell colonies were picked from the plates to obtain unialgal cell cultures. For all further experiments, the isolated diatoms were grown for 14 days in batch cultures in liquid f/2 medium in the same culture conditions as described above. Live cells were photographed in counting chambers using a Nikon TS300 inverted microscope (Nikon Corporation, Tokyo, Japan) equipped with a 100× PlanApochromatic oil immersion lens (n.a. = 1.40) and differential interference contrast (DIC) optics.

For light microscopy (LM) and electron microscopy (EM) observations, samples were cleaned of organic material by boiling the cultured cell suspension in a few tens of millitres of 30% hydrogen peroxide for a few hours, followed by addition of ca. 10 ml of 10% HCl to remove the calcium carbonate. After oxidation, cleaned samples were repeatedly rinsed with deionized water. Diatom suspension was then pipetted onto ethanol-cleaned cover slips and left to dry in the air. Naphrax (Brunel Microscopes Ltd, Wiltshire, U.K.) was used as a mounting medium for LM observations of cleaned material, which were conducted with a Zeiss Axioscope (Carl Zeiss, Jena, Germany) using phase contrast (PhC) and DIC with a 100× oil immersion objective (n.a. = 1.40).

Ultrastructural analysis was made with scanning and transmission electron microscopy (SEM and TEM, respectively). For SEM examination, a drop of the cleaned sample was filtered onto Whatman Nuclepore polycarbonate membranes (Fisher Scientific, Schwerte, Germany). Filters were air-dried overnight, mounted onto aluminum stubs, coated with gold-palladium or osmium. SEM observations were made at the Goethe University in Frankfurt am Main using a Hitachi S-4500 (Hitachi, Tokyo, Japan). SEM and TEM observations were done at the Warsaw University of Technology, Faculty of Materials Science and Engineering, using a Hitachi SEM/STEM S-5500, in which the specimens were simultaneously observed in scanning and transmission mode. As the newly described species has a very small size, we have supplemented our own LM characteristics with a few images kindly taken by ing. Wulff Herwig using his advanced light photomicrography system, for which a detailed description is presented at http://www.microscopy-uk.org.uk/mag/artmar11/Advanced_Light_Photomicrography.pdf. ([19]).

### Auxosporulation

Auxosporulation was studied in a *Biremis* sp. obtained in January and March 1990 from the sublittoral of Loch Goil at 56° 7.7′ N, 4° 54.2′ W, ca. 5 km from Lochgoilhead, Argyll, W Scotland. Details of the collection site are given by ([20]) and ([21]). Samples were collected manually by *sub aqua* divers from fine silty sand at 9–12 m below mean low water, spring tides, and epipelon was harvested using 24 × 50 mm cover slips to trap diatoms moving up towards the light through lens tissue placed on sediment from which the excess water had been removed by suction (after the samples had stood for several hours). The cover-slips and trapped epipelon were then incubated for up to three weeks (in the laboratory or an incubator) in enriched seawater medium, as described by ([21]). For incubations of more than a week, the medium was changed every 3–5 days.

Auxosporulation occurred after a week or more of incubation and was examined *in situ* on cover-slips incubated as above, after removing and mounting the slips on enriched seawater medium on a microscope slide; each cover-slip was ringed with Vaseline to prevent evaporation during a few hours of observation. Vegetative and auxosporulating cells were photographed on Kodak Technical Pan film (Estar base), using a Reichert Polyvar photomicroscope (100× objective, n.a. 1.32), or drawn with the aid of the Polyvar's drawing attachment. Selected line drawings were used by Mann ([22], Fig. 13–15) in a publication reviewing patterns of sexual reproduction in diatoms and so are not included here. The terminology of sexual reproduction and auxospores follows ([23]).

### DNA extraction, amplification and sequencing

Genomic DNA was isolated from 50 ml of a two-week-old algal cell culture using the GeneElute Plant Genomic DNA Miniprep Kit (Sigma–Aldrich, Hamburg, Germany) according to the manufacturer's instructions. The nuclear gene (SSU) and two chloroplast-encoded gene (*rbc*L, *psb*C) were amplified from genomic DNA using the proof reading polymerase Phusion (Finnzymes, Thermo Scientific, Schwerte, Germany) following the PCR protocol as described in ([24], [25]). The primers used for amplification are listed in Table 1. PCR products were visualized in a 1% agarose gel and then purified using Exo/Sap enzyme mixture (Thermo Scientific Fermentas) and sent to oligo.pl DNA Sequencing Laboratory IBB PAS, Warsaw, Poland for Sanger sequencing with use of BigDye Terminator v. 3.1 chemistry and ABI3730 xl sequencer. For *rbc*L, purified PCR product has been sent to MWG Operon (now Eurofins Genomics Ebersberg, Germany) for sequencing on ABI 3730 xl machine.

**Table 1:** Primers used to amplify the SSU, *rbc*L and *psb*C genes.

Name	Gene	Suquence (5′-3′)	Reference
psbC-F	*psb*C	CAC GAC CWG AAT GCC ACC AAT	[24]
psbC-R	*psb*C	ACA GGM TTY GCT TGG TGG AGT GG	[24]
SSU-F	SSU	AAC CTG GTT GAT CCT GCC AGT	[24]
ITS1DR^2^	SSU	CCT TGT TAC GAC TTC ACC TTC C	[24]
DPrbcL1	*rbc*L	AAGGAGAAATHAATGTCT	[25]
DPrbcL7	*rbc*L	AARCAACCTTGTGTAAGTCTC	[25]

The *rbc*L sequences for *Neidium* sp. NEI323TM, *Neidium* sp. NEI 44, *Neidium* sp. NEI428T and *Neidium* sp. NEI Baik482, and *Biremis* sp. were obtained by G.E. Simpson, M. Hollingsworth and A. Clark as described in ([25]).

### Phylogenetic analysis

Analysis of the three-gene (SSU, *rbc*L and *psb*C) dataset was performed using 73 diatom taxa (Table 2), using *Ctenophora pulchella* (Ralfs ex Kützing) D.M. Williams & F.E. Round and *Tabularia* cf. *tabulata* (C. Agardh) Snoeijs as the outgroups. GenBank accession numbers for the sequences used can be found in Table 2. Sequences were aligned by eye using Mesquite version 2.75; alignments are available in Appendix S1. Prior to phylogenetic analysis, the SSU rDNA inserts of *B. panamae* were excluded and the terminal ends of all sequences were trimmed to have the same length (aligments with *B. panamae* inserts are included in Appendix S2). The data were partitioned by gene and by codon position (in the case of the chloroplast markers) with a GTR+G+I model. Maximum Likelihood (ML) analyses were run using RAxML v. 7.2.6 ([26]). The analysis consisted of multiple runs (100), each with 1000 bootstrap replicates and the tree with the best log likelihood score was chosen as our maximum likelihood estimate (see results section).

**Table 2:** GenBank accession of SSU rDNA, rbc*L* and psb*C* sequences derived from the species used in the phylogenetic analysis.

Species	Strain	Genebank Accession		
		SSU	*rbc*L	*psb*C
*Achnanthes* sp.	SanNicAchnan	KC309473	KC309545	KC309617
*Achnanthes* sp.	ECT3684	KC309476	KC309548	KC309620
*Achnanthes* sp.	ECT3911	KC309475	KC309547	KC309619
*Achnanthes coarctata* Brébisson ex W. Smith	UTEX FD185	HQ912594	HQ912458	HQ912287
*Bacillaria paxillifer* (O.F. Müller) T. Marsson	UTEX FD468	HQ912627	HQ912491	HQ912320
*Berkeleya rutilans* (Trentepohl ex Roth) Grunow	ECT3616	HQ912637	HQ912501	HQ912330
*Biremis panamae* Barka, Witkowski, Weisenb.	P136	**KM078661**	**KM078662**	**KM078668**
*Biremis* sp.	RhoB2	–	**KM078667**	–
*Caloneis lewisii* Patrick	UTEX FD54	HQ912580	HQ912444	HQ912273
*Campylodiscus clypeus* (Ehrenberg) Ehrenberg ex Kützing	L951	HQ912412	HQ912398	HQ912384
*Campylodiscus* sp.	3613.8	HQ912413	HQ912399	HQ912385
*Climaconeis riddleae* A.K.S.K. Prasad	ECT3724	HQ912644	HQ912508	HQ912337
*Cocconeis placentula* Ehrenberg	UTEX FD23	HQ912592	HQ912456	HQ912285
*Cocconeis* sp.	ECT3901	KC309479	KC309551	KC309622
*Cocconeis stauroneiformis* (W. Smith) Okuno	s0230	AB430614	AB430694	–
*Craticula cuspidata* (Kutzing) D.G. Mann	UTEX FD35	HQ912581	HQ912445	HQ912274
*Ctenophora pulchella* (Kützing) D.M. Williams & F.E. Round	UTEX FD150	HQ912611	HQ912475	HQ912304
*Cylindrotheca closterium* (Ehrenberg) Reimann & J. Lewin	CCMP1855	HQ912645	HQ912509	HQ912338
*Cymatopleura elliptica* (Brébisson) W. Smith	L1333	HQ912659	HQ912523	HQ912352
*Denticula kuetzingii* Grunow	UTEX FD135	HQ912610	HQ912474	HQ912303
*Diploneis subovalis* Cleve	UTEX FD282	HQ912597	HQ912461	HQ912290
*Entomoneis ornata* (Ehrenberg) Ehrenberg	14A	HQ912411	HQ912397	HQ912383
*Entomoneis* sp.	CS782	HQ912631	HQ912495	HQ91232
*Epithemia argus* (Ehrenberg) Kützing	CH211	HQ912408	HQ912394	HQ912380
*Epithemia sorex* Kützing	CH148	HQ912409	HQ912395	HQ912381
*Epithemia turgida* (Ehrenberg) Kützing	CH154	HQ912410	HQ912396	HQ912382
*Eunotia bilunaris* (Ehrenberg) Schaarschmidt	UTEX FD412	HQ912599	HQ912463	HQ912292
*Eunotia glacialis* Meister	UTEX FD46	HQ912586	HQ912450	HQ912279
*Eunotia pectinalis* (Kützing) Rabenhorst	NIES461	HQ912636	HQ912500	HQ912329
*Eunotia* sp.	ECT3676	KC309480	KC309552	KC309623
*Fallacia monoculata* (Hustedt) D.G. Mann	UTEX FD254	HQ912596	HQ912460	HQ912289
*Fallacia pygmaea* (Kützing) A.J. Stickle & D.G. Mann	UTEX FD294	HQ912605	HQ912469	HQ912298
*Fistulifera pelliculosa* (Brébisson) Lange-Bertalot	CCMP543	–	HQ337547	–
*Fistulifera saprophila* (Lange-Bertalot & Bonik) Lange-Bertalot	TCC508	KC736618	KC736593	–
*Gomphonema affine* Kützing	UTEX FD173	HQ912608	HQ912472	HQ912301
*Gomphonema parvulum* (Kützing) Kützing	UTEX FD241	HQ912595	HQ912459	HQ912288
*Gyrosigma acuminatum* (Kützing) Rabenhorst	UTEX FD317	HQ912598	HQ912462	HQ912291
*Halamphora coffeaeformis* (C. Agardh) Levkov	7977-AMPH101	KJ463449	KJ463479	KJ463509
*Hantzschia amphioxys* var. *major* Grunow	A4	HQ912404	HQ912390	HQ912376
*Lemnicola hungarica* (Grunow) F.E. Round & P.W. Basson	UTEX FD456	HQ912626	HQ912490	HQ912319
*Mayamaea permitis* (Hustedt) K. Bruder & L.K. Medlin	TCC540	KC736630	KC736600	–
*Meuniera membranacea* (Cleve) P.C. Silva in Hasle & Syvertsen	ECT3896	KC309482	KC309554	KC309624
*Navicula cari* Ehrenberg	AT-82.04	AM501991	AM710457	–
*Navicula cryptocephala* Kützing	UTEX FD109	HQ912603	HQ912467	HQ912296
*Navicula reinhardtii* Grunow	AT-124.15	AM501976	AM710442	–
*Navicula tripunctata* (O.F.Müller) Bory de Saint-Vincent	AT-202.01	AM502028	AM710495	–
*Neidium affine* (Ehrenberg) Pfizer	UTEX FD127	HQ912583	HQ912447	HQ912276
*Neidium bisulcatum* (Lagerstedt) Cleve	UTEX FD417	HQ912591	HQ912455	HQ912284
*Neidium productum* (W. Smith) Cleve	UTEX FD116	HQ912582	HQ912446	HQ912275
*Neidium* sp.	NEI44	–	**KM078663**	–
*Neidium* sp.	NEIBaik482	–	**KM078664**	–
*Neidium* sp.	NEI323TM	–	**KM078665**	–
*Neidium* sp.	NEI428T	–	**KM078666**	–
*Nitzschia dubiiformis* Hustedt	s0311	AB430616	AB430696	–
*Nitzschia filiformis* (W. Smith) Hustedt	UTEX FD267	HQ912589	HQ912453	HQ912282
*Phaeodactylum tricornutum* (Brébisson) W. Smith	CCMP2561	HQ912556	HQ912420	HQ912250
*Pinnularia brebissonii* (Kützing) Rabenhorst	UTEX FD274	HQ912604	HQ912468	HQ912297
*Pinnularia termitina* (Ehrenberg) R.M. Patrick	UTEX FD484	HQ912601	HQ912465	HQ912294
*Placoneis elginensis* (Gregory) E.J. Cox	UTEX FD416	HQ912607	HQ912471	HQ912300
*Psammodictyon constrictum* (Gregory) D.G. Mann	s0309	AB430617	AB430697	–
*Rhopalodia contorta* Hustedt	L1299	HQ912406	HQ912392	HQ912378
*Rhopalodia gibba* (Ehrenberg) O. Müller	CH155	HQ912407	HQ912393	HQ912379
*Rhopalodia* sp.	9vi08.1F.2	HQ912405	HQ912391	HQ912296
*Rossia* sp.	E3333	EF151968	EF143281	–
*Scoliopleura peisonis* Grunow	UTEX FD13	HQ912609	HQ912473	HQ912302
*Sellaphora capitata* D.G. Mann & S.M. McDonald	BLA11	–	EF143316	–
*Sellaphora pupula* (Kützing) Mereschkowsky	BLA14	–	EF143294	–
*Stauroneis acuta* W. Smith	UTEX FD51	HQ912579	HQ912443	HQ912272
*Stenopterobia curvula* (W. Smith) Krammer	L541	HQ912416	HQ912402	HQ912388
*Surirella minuta* Brébisson	UTEX FD320	HQ912658	HQ912522	HQ912351
*Surirella splendida* (Ehrenberg) Kützing	19C	HQ912415	HQ912401	HQ912387
*Tabularia* cf. *tabulata* (C. Agardh) Snoeijs	CCMP846	HQ912615	HQ912479	HQ912308
*Tryblionella apiculata* Gregory	UTEX FD465	HQ912600	HQ912464	HQ912293

Sequences obtained in this paper are indicated in bold.

Two Bayesian Inference analyses, each with 3 chains, were run with MrBayes v.3.2 ([27]), using a 6-substitution model, partitioning the dataset by gene and by codon. Thirty million generations were run per analysis and all but the final million generations were discarded as “burn-in”; the remaining trees were compared in Tracer v.1.5 ([28]) for evidence of convergence. These final 10,000 trees were used to generate a majority rule consensus tree and obtain posterior probabilities for nodes (see results ection).

### Nomenclature

The electronic version of this article in Portable Document Format (PDF) in a work with an ISSN or ISBN will represent a published work according to the International Code of Nomenclature for algae, fungi, and plants, and hence the new names contained in the electronic publication of a PLOS ONE article are effectively published under that Code from the electronic edition alone, so there is no longer any need to provide printed copies. The online version of this work is archived and available from the following digital repositories: PubMed Central and LOCKSS.

## Results

### Taxonomic treatment

Order: Naviculales

Suborder: Neidiineae

Family: Scoliotropidaceae

*Biremis panamae* Barka, Witkowski & Weisenborn, sp. nov.

Fig. 2A-O

**Figure 2:**
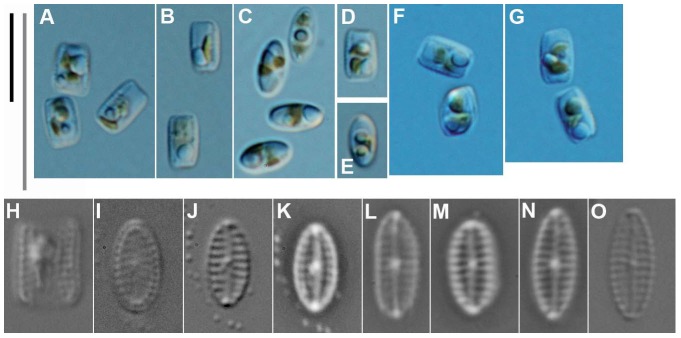
*Biremis panamae* sp. nov., living cells and cleaned valves under LM. **A–G**. Living specimens photographed from the clonal culture. Note the two chloroplasts per cell. **H–O**. Cleaned material from the clonal culture. Fig. 2H–J were taken with advanced photomicroscopy system; Fig. 2K–N. were taken with phase contrast optics; Fig. 2O. was taken with differential interference contrast (DIC). Fig. 2H is a frustule in girdle view; Fig. 2I–O are valves in valve view. [Scale bars  =  10 µm; the grey bar only for Figures H–O].

**Figure 3:**
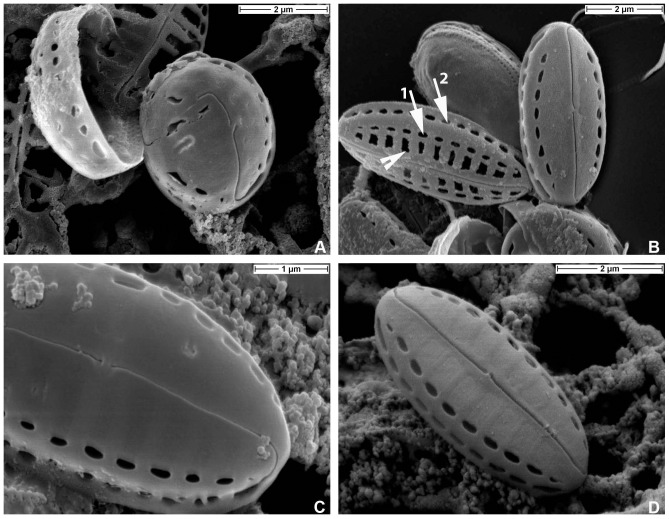
*Biremis panamae* sp. nov., SEM: external valve views. **A.** Two valves of exceptionally small specimens after size reduction in culture, showing teratological development of the raphe. **B.** Two well developed specimens, one eroded and showing the valve face striae with destroyed occlusions. Arrowhead points to corroded occlusions of the valve face striae. Note also two rows of foramina, one on the valve face (arrow 1) and the other on the valve mantle (arrow 2). **C.** Detail of a valve with intact pore occlusions, showing the external central raphe endings slightly bent towards the valve secondary side and strongly bent apical raphe end, which terminates in one of the foramina positioned on the valve mantle. **D.** Fully intact, uneroded valve, showing the valve face areolae completely closed with hyaline occlusions (hymenes), which are slightly depressed below elevated interstriae (virgae).

#### Description

Frustules small, rectangular to almost square in girdle view with broadly rounded corners. The girdle fairly broad, girdle bands in LM barely distinguishable (**Fig. 2H**). Plastids plate-like, two per cell (sometimes only one?), arranged diagonally, or with one towards each apex (**Fig. 2A–G**). Valves elliptical to linear elliptical with broadly rounded apices, 4.0–7.0 µm long and 2.0–2.6 µm wide. Axial area barely distinguishable, linear; central area distinguishable. Raphe straight, filiform, with external central endings very close to each other; apical endings not observable in LM (Fig. 2I–O). Transapical striae distinct, 17–20 in 10 µm, in the middle parallel, becoming slightly radiate towards apices. With bright-field optics, valves appear like a tiny *Navicula*; however, with phase contrast optics, the chambered nature of the striae is recognizable (**Fig. 2K**). The complex nature of the valve structure is also detectable with advanced light microphotography, where the rows of marginal areolae can be observed in valve view (**Fig. 2I**) and especially in girdle view (**Fig. 2H**).

#### Valve exterior in EM. Fig. 3A–D, Fig. 4A–D, Fig. 5A–D

**Figure 4:**
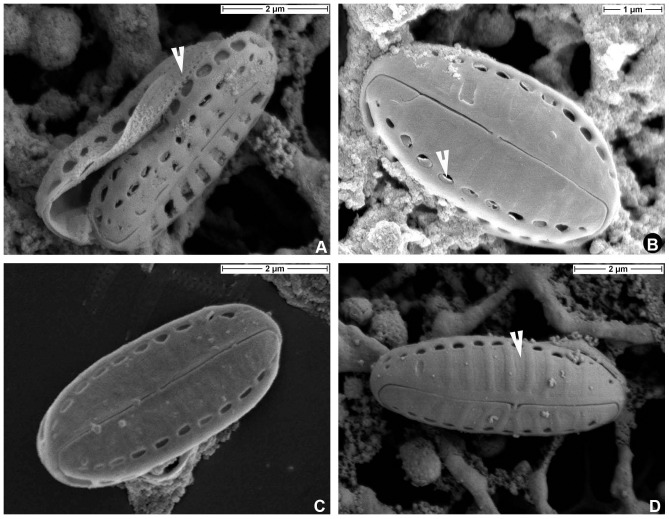
*Biremis panamae* sp. nov., SEM: external valve views. **A.** Frustule with detached valves and a part of a copula with several rows of pores (arrowhead). **B.** Valve face showing occlusions of the marginal row of small areolae (arrowhead). **C, D.** External valve view showing variation in the valve face morphology. Note the depressed surface of the valve face areola occlusions (arrowhead).

**Figure 5:**
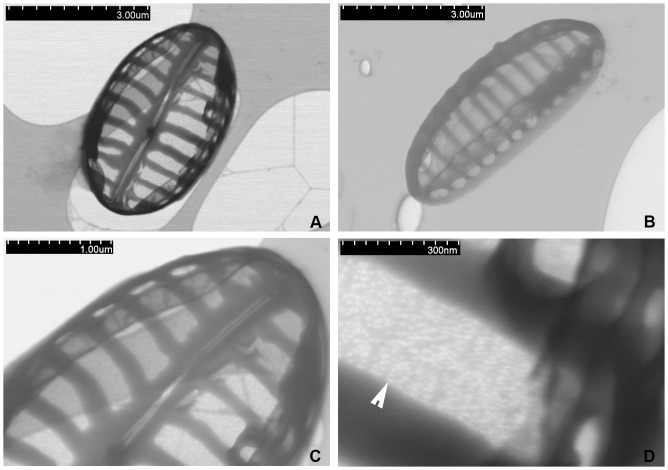
*Biremis panamae* sp. nov., TEM. A. A whole valve in valve view. **B.** A whole specimen observed from the valve interior. **C, D.** Close ups of a specimen illustrated in Fig. 5A; note the finely porous areolae occlusions, arrowhead in Fig. 5D.

The valve face is slightly but distinctly domed. The transition between valve mantle and valve face is fairly abrupt and is marked by a narrow hyaline strip, which interrupts the striae and separates them into two zones: (1) a complex zone on the valve face differentiated into (A) a row of large transapically elongate, strip-like areolae located adjacent to the raphe, in the proximal part of the valve face, and (B) a row of apically elongate, elliptical foramina positioned along the edge of the valve face; and (2) a narrow strip on the valve mantle containing a second row of apically elongate foramina adjacent to the valve margin (**Fig. 3B, D** **,** **Fig. 4A, B**). The axial area is narrow and linear and the central area very small and asymmetrical, comprising only a slight shortening of a central stria on the valve's secondary side. The raphe-sternum is slightly elevated above the general valve surface. The raphe is filiform, straight to slightly bent, with the external central endings very close to each other, slightly expanded, and straight or bent slightly towards the secondary side of the valve. The external apical raphe endings are strongly hooked in the same direction and terminate on the valve mantle in a small groove in line with the row of mantle foramina (**Fig. 3A, B** **,** **4A–C**). The girdle is composed of several copulae, each finely perforated (**Fig. 4A**). TEM observations show that the transapically elongate areolae of the proximal part of the valve face are covered by a delicate membrane of porous silica with tiny round holes resembling the hymenate occlusions of other raphid diatoms ([29]). This fine membrane, which lies slightly below the remainder of the valve face externally, is lost in eroded specimens, revealing the shape of the areolae and also that the areolae are wider than the virgae (transapical ribs, **Fig. 3B** **,** **Fig. 4A, B** **,** **Fig. 5A–D**).

#### Valve interior in EM

**Figure 6:**
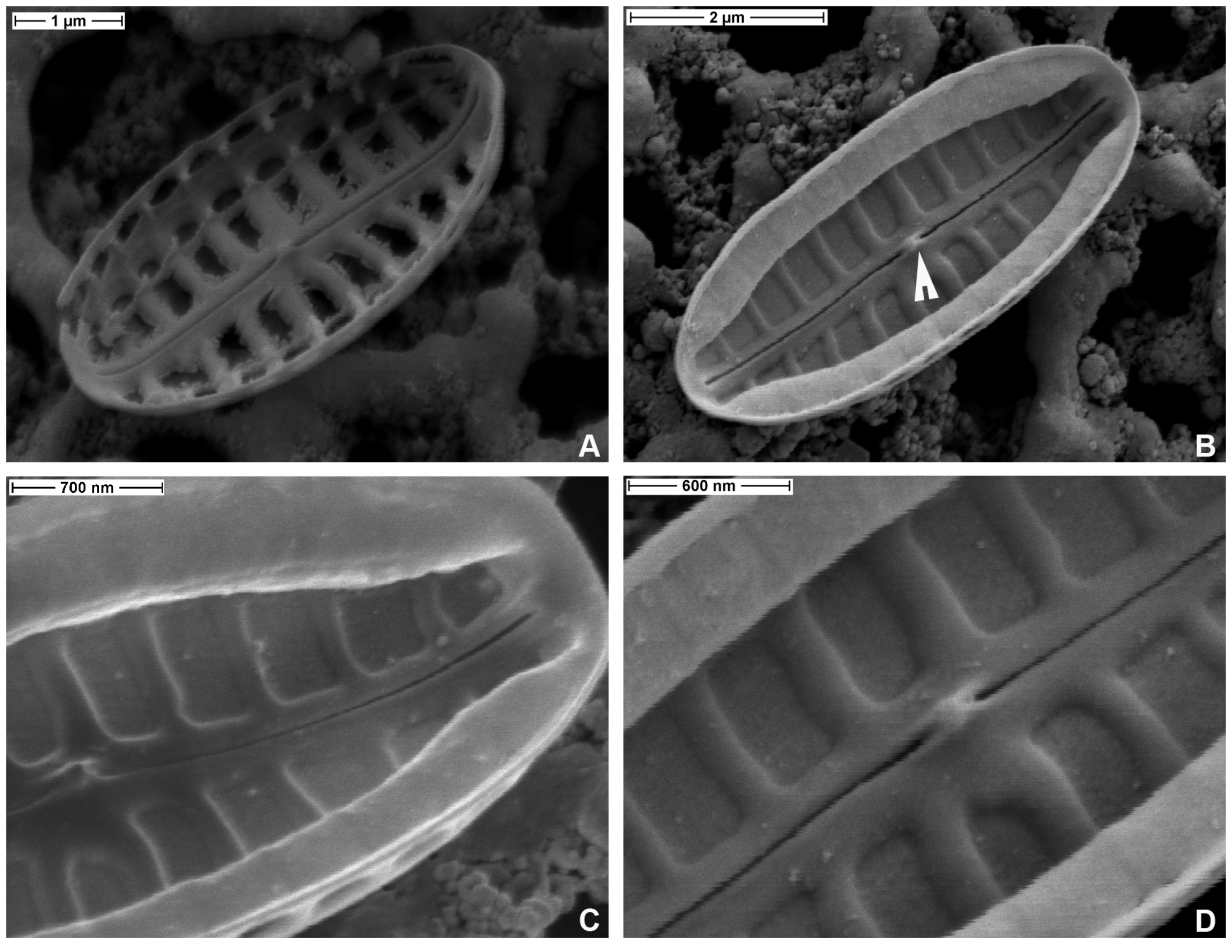
*Biremis panamae* sp. nov., SEM: internal valve views. **A.** Specimen with corroded chambers. **B.** An uneroded specimen with well preserved chambers; note the presence of a double helictoglossa (arrowhead) between the central raphe endings. **C, D.** Close up of the specimen illustrated in Fig. 6B: note the narrow elevated virgae, separated by depressed areolae.

**Fig. 6A–D**.The valve surface is flat internally, with depressed striae separated by raised virgae, except at the side of the valve, where there is a line of distinct chambers forming a tubular structure. The tube of chambers extends almost to the valve apex. Each chamber is aligned with a stria (see the eroded specimens in **Fig. 6A** and the complete specimen in **Fig. 6B**) and eroded specimens (**Fig. 6A**) show that it extends from the edge of one valve face areola, beneath the hyaline strip at the margin of valve face and mantle, to the valve margin; it opens to the exterior by two foramina (compare **Fig. 4A–C**). In other *Biremis* species, the interior wall of the chambers is porous (e.g. [1]) but we did not detect pores in our material. The raphe-sternum is somewhat elevated internally and the raphe slit is straight and simple. The internal central endings are simple and coaxial and terminate in a ‘double helictoglossa’, i.e. a slightly elongate, almost beak-like mass of silica capping the raphe slits. Likewise, the apical internal endings terminate in a very small and simple helictoglossa (**Fig. 6A–D**).

HOLOTYPE: slide no. SZCZ 18710A housed in the diatom collection of Andrzej Witkowski, at the Palaeoceanology Unit of Faculty of Geosciences at the University of Szczecin (SZCZ), leg. Frederik Barka Jan. 20th 2011.

ISOTYPES: Coll. Lange-Bertalot (FR) SEM stub B711; Hustedt Collection AWI Bremerhaven slide no. ZU9/92.

TYPE HABITAT: The diatom was collected from the drift line of the Pacific Ocean on "Playa Monagre" beach in Panama in the Los Santos Province (7° 58′ 32.46″ S and 80° 20′ 50.31″ W).

DERIVATION OF NAME: The specific name refers to the name of the country of sampling, Panama.

### Comparison with established taxa

Most of the *Biremis* species described from marine or brackish habitats are much larger than *B. panamae* and could not be confused with it, likewise the freshwater taxa. The only similar species in terms of size is *B. lucens*, though its valves are always longer and broader (7.6–25.5 µm long and 2.5–5.6 µm wide) and the striae are coarser (11–17 in 10 µm, as opposed to 17–20 in *B. panamae*). Furthermore, *B. lucens*, like most other marine *Biremis* taxa, has transapical striae that are composed only of the marginal chambers, with their two rows of external foramina ([10]): there are no areolae in the central zone of the valve face. *Biremis panamae*, on the other hand, along with the two rows of foramina (one along the margin of the valve face, the other on the valve mantle), has an areolated valve face with areolae that are closed by delicate porous membranes (hymenate occlusions, detected in TEM: **Fig. 5D**).

### Auxosporulation in *Biremis* sp. Fig. 7A–J

**Figure 7:**
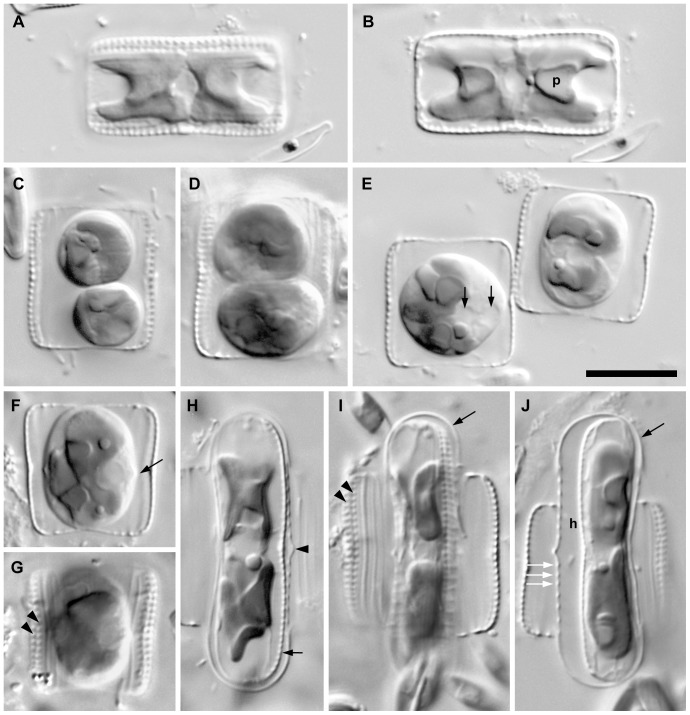
Vegetative cells and auxosporulation in *Biremis* sp. A, B. Two focuses of a vegetative cell in girdle view. Each cell contains two chloroplasts either side of the centre, each of which comprises two plates (one is shown for each chloroplast in Fig. 7A, the other being out of focus beneath, on the opposite side of the cell) connected by a narrow bridge containing the pyrenoid (e.g. p). **C, D.** Two paired gametangia, each containing two rounded, rearranged gametes. The gametangia were paired with their girdles adjacent, the cell shown in Fig. 7D lying immediately below that in Fig. 7C. **E**. Two paired gametangia, unusual in being in contact only via their valves. Each gametangium contains a single subspherical zygote. Two nuclei are visible in the left-hand cell (arrows) and two of the four chloroplasts in the right-hand cell. **F, G.** Two focuses of a gametangium containing a zygote on the point of transformation into an auxospore. Note the slight central inflection of the zygote's outline, marking the deposition of the primary transverse perizonial band (cf. Fig. 7H, arrowhead). The two rows of foramina on the valves can be seen in Fig. 2G (arrowheads). **H**. Expanded auxospore containing the initial epivalve (in section at arrow). The auxospore is encased in a well developed perizonium, containing a primary transverse band flanked by several secondary bands (see in section: see also Figs 7I, J). **I.** Peripheral focus of an expanded auxospore containing the initial epivalve. The two rows of foramina on one of the gametangium valves can be seen (arrowheads). The end of the auxospore is covered by a siliceous cap (arrow). **J.** Expanded auxospore containing a completed initial cell. The initial hypovalve (in section at h) lies at a distance from the perizonium, as a result of a strong contraction of the protoplast immediately before its formation; the initial epivalve lies opposite, directly moulded by the interior of the perizonium. The auxospore casing can be seen to consist of a perizonium of transverse bands (e.g. at white arrows) and two silicified hemispherical caps (e.g. at black arrow). [Scale bar 10 µm].

In order to help place *Biremis* phylogenetically and taxonomically, we studied auxosporulation in an unnamed *Biremis* species (**Fig. 7**). Cells almost always lay in girdle view (e.g. **Fig. 7A, B**) because the valves were much narrower than the girdle. Valves had marginal chambers and two rows of external foramina (visible in **Fig. 7G, I**, as in *B. panamae*). Living cells contained two chloroplasts, one towards each end of the cell (**Fig.7A, B**). Each chloroplast comprised two X- or butterfly-shaped plates, one lying against each side of the girdle (**Fig. 7A**), connected by a narrow bridge containing a compact, ±isodiametric pyrenoid (**Fig. 7B**). In valve view each chloroplasts resembled a narrow ‘H’ (not illustrated), with the pyrenoid occupying the cross-bar.

Prior to sexual reproduction, cells became paired, usually lying with their girdles adjacent, and entered meiosis. **Fig. 7C and D** are two focuses (C above, D below) of a single pair of cells (gametangia) that had completed meiosis I and produced two gametes per cell. In the pair shown in **Fig. 7E**, on the other hand, the cells lay displaced from each other, with only the valves touching. The gametes were formed by cleavage of the gametangium parallel to the valves after meiosis I (not shown: see [22], Fig 13). Subsequently, the gametes rounded up and swiveled around to lie one towards each end of the gametangium (**Fig. 7C, D**). During fertilization, one gamete moved out from its gametangium and into the other gametangium, so that finally each gametangium contained a single subspherical zygote, which contained two nuclei (**Fig. 7E**, left), the other two nuclei from meiosis having previously degenerated, and also four chloroplasts (see also [22], Fig. 13–15). There was then a phase during which the zygote matured, becoming elliptical and acquiring a robust wall, and transformed into an auxospore. The first stage of auxospore expansion could be detected through the appearance of the primary transverse perizonial band around the equator of the cell (**Fig. 7F**, arrow; cf. **Fig. 7H**). Auxospore expansion took place parallel to the long (apical) axis of the gametangium (**Fig. 7I, J**) and was accompanied by formation of further transverse perizonial bands, clearly visible as periodic thickenings of the auxospore wall (e.g. white arrows, **Fig. 7J**). During expansion it became obvious that the original wall of the zygote (comprising the incunabula sensu Kaczmarska et al. 2013 ([23]) had been composed of two smooth, evenly curved hemispheres (e.g. black arrows, **Fig. 7I, J**). These survived oxidation (by burning) to remove organic material, revealing that they were strongly silicified, like the valves, girdle bands and perizonial bands.

Once expansion was complete, the initial epivalve was formed within the perizonium, beneath one of the valves of the gametangium. The initial epivalve was closely appressed to the perizonium over its whole length, except at the centre, where there was a slight contraction (**Fig. 7H**). In contrast, the initial hypovalve was formed after a marked contraction of the cell away, so that there was a wide space between it and the perizonium (**Fig. 7J**). Expanded auxospores were ca. 30.0–32.0 µm long and gametangia 14.0–18.0 µm.

### Phylogeny

**Figure 8:**
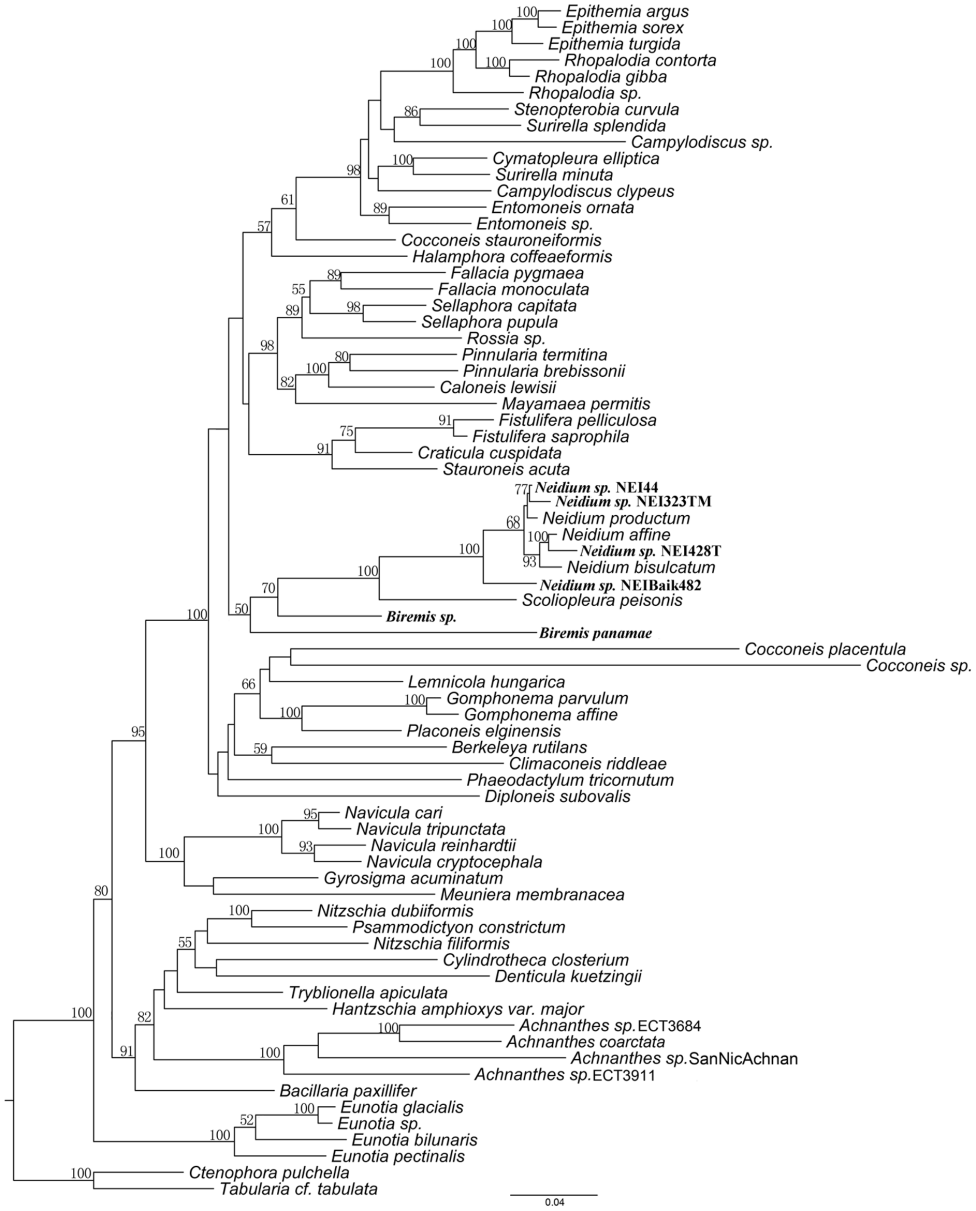
Maximum likelihood phylogeny (with bootstrap values at nodes) inferred from a concatenated alignment of *rbc*L, *psb*C and SSU markers. *Neidium* sp. NEI323TM, *Neidium* sp. NEI 44, *Neidium* sp. NEI428T and *Neidium* sp. NEI Balk482 represent previously unpublished *rbc*L gene sequences from different *Neidium* species. *Biremis* sp. represents a *rbc*L gene sequence from an unpublished *Biremis* sp. The tree is rooted with the pennate araphid taxa *Ctenophora pulchella* and *Tabularia* cf. *tabulata*. Support values lower than 50% were not included in the tree. The GenBank *Achnanthidium coarctatum* name has been changed to *Achnanthes coarctata*.

The size of the amplified *B. panamae* SSU rDNA gene was found to be ca. 3300 bp, whereas we expected its size to be ca. 1700 bp, based on the published sequences of other raphid diatoms. Through alignment of our SSU rDNA sequences to existing SSU sequences of diatoms, we discovered the presence of 12 inserts with a total length of ca. 1500 bp (Appendix S2). In a three-gene maximum likelihood (ML) and Bayesian Inference (BI) phylogenetic analysis (**Fig. 8** **,** **9** respectively) *Biremis* appeared most closely related to *Neidium* and *Scoliopleura*. The two *Biremis* spp. formed a grade, with *Biremis* sp. sister to the *Scoliopleura*–*Neidium* clade (with low support: 70% ML bootstrap, 0.73 BI posterior probability) and *B. panamae* sister to the *Biremis* sp.–*Scoliopleura–Neidium* assemblage (again with rather low support: 50% bootstrap, 0.99 posterior probability). As for the relationship of the *Biremis–Scoliopleura–Neidium* clade to the rest of the raphid pennate diatoms, not much can be said at this point. In both ML and BI analyses, the *Biremis* clade was in an unresolved trichotomy sister to the *Navicula* clade (ML; 95% bootstrap) or *Diploneis* (BI; 1.00 posterior probability). It should be noted that *Biremis* sp. is represented by a single *rbc*L sequence, which likely affects the support values in the *Biremis* clade and may explain why the two *Biremis* strains do not form a clade. However, when we performed a RAxML analysis based only on the *rbc*L alignment the topology of the *Biremis–Scoliopleura–Neidium* clade was almost identical to that in the three gene tree (the *rbc*L tree is included in the supplementary data), with *Biremis panamae* sister to the *Scoliopleura–Neidium* clade and *Biremis* sp. sister to that clade. The bootstrap support was lower than that in the three-gene tree (less than 50, Appendix S3).

**Figure 9:**
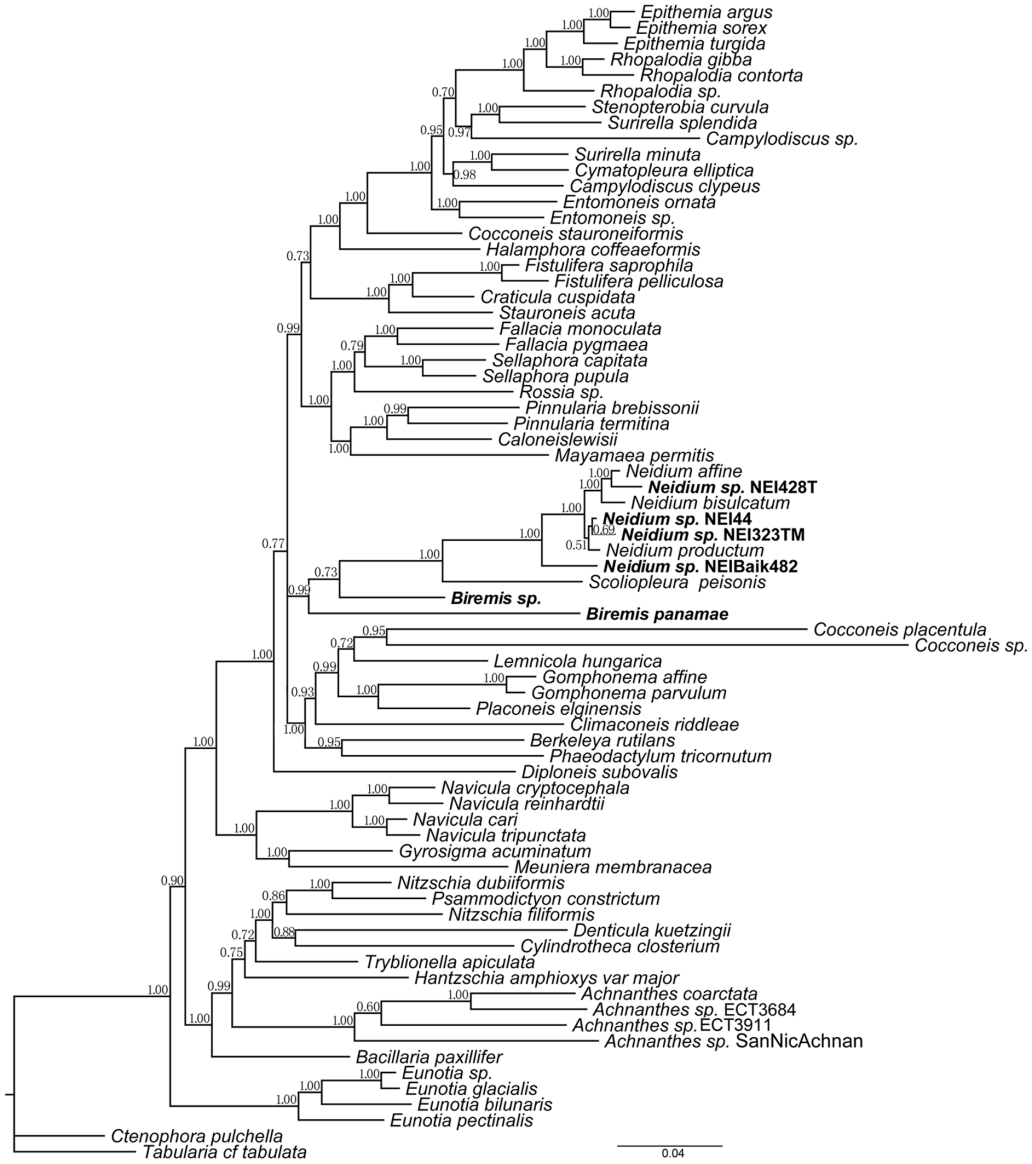
Bayesian Inference phylogeny inferred from a concatenated alignment of *rbc*L, *psb*C and SSU markers. Posterior probabilities are shown at the nodes. The tree is rooted with the araphid pennate taxa *Ctenophora pulchella* and *Tabularia* cf. *tabulata*.

## Discussion

Superficially, *B. panamae* looks in LM like one of the numerous tiny *Navicula* species found in fresh and marine waters. Only the high magnification LM images (**Fig. 2H–N**) taken with a phase contrast or examination with EM reveal the true structure of the valve and identify the species as belonging to *Biremis*. The most characteristic feature is the stria structure, with aligned chambers forming a segmented tube located along the valve mantle (cf. [1], [2], [10]). The difference between the generitype (*B. ambigua*) and *B. panamae* is the presence of large, transapically elongate areolae on the valve face of *B. panamae*, lying between a row of apically elongate foramina near the margin of the valve face (which open into the marginal chambers) and the raphe-sternum. Until now, two types of valve face have been observed in marine *Biremis*: in one the valve face is plain (*B. ambigua* and *B*. *lucens*: [1], [2], [10]), while in the second the valve face is striated (e.g. [2], [11]). The *Biremis* species in which we observed auxosporulation belongs to the first type (our unpublished observations), whereas *B. panamae* clearly belongs to the second type, which was previously known only in an unnamed *Biremis* species ([2], Fig. 3: 22, 24, 26), *B. solitaria*, and *Biremis* spec. 155/2 from the Gulf of Gdańsk (the Baltic Sea: [11], Fig. 154: 17, 18 and 155: 8 respectively). Because of the striated valve face, *B. panamae*, *B. solitaria* and the two unnamed *Biremis* species studied by Cox (1990 [2]) and Witkowski et al. (2000, [11]) seem to provide a link between *Biremis* and the freshwater genus *Pulchella*, which was erected by Krammer (2000, [30]) for two species previous assigned to *Biremis*, namely *B. schwabei* (Krasske) Lange-Bertalot in Lange-Bertalot, Külbs, Lauser, Nörpel-Schempp & Willmann and *B. kriegeriana* (Krasske) Lange-Bertalot in Lange-Bertalot, Külbs, Lauser, Nörpel-Schempp & Willmann. *Pulchella kriegeriana* possesses punctate striae on the valve face ([30], Fig. 1e), whereas *P. schwabei* does not ([30], Fig. 9–17) and thus resembles the freshwater *Biremis* species described by Vyverman et al. (1997, [9]). The distinction, if any, between these two genera needs further study.

The marginal chambers of *B. panamae* are very similar in their general features (chambers closed internally by a delicate membrane that bulges into the cell interior; opening externally via two large foramina, which are aligned to form two longitudinal rows on each side of the valve) to the chambers seen in other *Biremis* and also to the more elaborate and longer chambers present in *Scoliopleura*, *Scoliotropis* and *Progonoia* ([1]). In all too, the raphe terminates internally in a double helictoglossa and there are either two H-shaped chloroplasts as in *Biremis* (**Fig. 7A, B**), or four chloroplasts, like the four plate-like halves of the two chloroplasts of *Biremis*, but without the pyrenoid connection between them.

*Biremis* was established as a genus quite recently ([1]) and currently contains only ca. 17 species (see Introduction), some described as new but most transferred from *Pinnularia*, *Amphora*, or *Navicula* [where they were described and illustrated by authors such as Cleve, Heiden, Hustedt and Giffen: ([3], [16], [4], [5], [6], [7]). The three gene based phylogeny resulting from our research shows, however, that *Biremis* is not closely related to *Pinnularia* or *Navicula*, nor to *Amphora* (which is not included in our tree but belongs close to the Rhopalodiales and Surirellales: [31], [32]). Instead, *Biremis* belongs in the same well supported clade as *Neidium* and *Scoliopleura*. The classification of *Biremis* and *Scoliopleura* in the same family (Scoliotropidaeae) was proposed without explanation by ([1]), but was based on the marginal chambering of the valve, chloroplast structure and double helictoglossa internally at the centre (D.G. Mann, unpublished information). In these respects, morphological observations and molecular data are consistent, supporting classification of both in the same family. However, in our tree, *Scoliopleura* is sister, not to *Biremis* but to *Neidium*, which argues against placing this *Neidium* in a separate family Neidiaceae (as done by [1]), if at the same time *Biremis* and *Scoliopleura* are placed together in the Scoliotropidaceae, because it would make the Scoliotropidaceae paraphyletic.

Although the link between *Biremis* and *Neidium* may initially look surprising, there are in fact several non-molecular (cytological, raphe and auxospore) characters that are consistent with a close relationship between them: this has been the subject of several studies in the last decades (e.g. [22], D.G. Mann and G.E. Simpson, unpublished). Morphological observations reveal that the frustule structure and habitat of *Neidium* and *Biremis* differ in numerous aspects. The major differences are chambered striae, curved polar raphe endings and simple external central raphe endings in *Biremis*, whereas in *Neidium* there is a longitudinal canal at the valve face periphery, forked apical raphe endings, and oppositely curved central raphe endings (e.g. [33], [1]). In addition, *Neidium* is an obligately freshwater genus, whereas *Biremis* spans marine and freshwaters. However, despite these contrasting characteristics, *Biremis* and *Neidium* are linked by the mode of auxosporulation (discussed further below) and a cytological character, namely bilineal transmission of chloroplasts through the mitotic cell cycle: because of the way the chloroplasts divide and are inherited (documented in *B. ambigua* by Cox 1990 [2] and in *Neidium* by Mann 1984 [34], 1996 [35]), each *Neidium* and *Biremis* cell possesses one or two individual organelles of each of two ‘clones’ of chloroplasts. Furthermore, both genera have a double helictoglossa internally (between the central raphe endings), and in them and also in *Scoliotropis*, *Scoliopleura* and *Progonoia* the valves have a two-layered structure over at least part of the valve, with fine internal pore occlusions internally and larger open pores or foramina externally ([1]). Neither bilineal transmission of chloroplasts nor the presence of a double helictoglossa is unique to *Neidium*, *Biremis* and related taxa, but they are unusual in raphid diatoms ([35], D.G. Mann unpublished observations) and tend to corroborate the molecular phylogeny.

With respect to auxosporulation, ([22]) has already pointed several similarities between *Biremis* and *Neidium*, especially the presence of ± hemispherical silicified caps over the ends of the auxospores, which are produced by the zygote before it begins to expand. The nature of the caps has since been investigated in detail by Mann & Poulíčková (2009 [36]; see also **Fig. 7H–J**). Although caps (derived from the incunabula of the zygote) occur on the poles of many pennate diatoms, they are usually either wholly organic (e.g. [37]) or contain small silica scales (e.g. [38], [39]): fully silicified unitary caps are known so far with certainty only in *Biremis* (this paper), *Neidium* ([36]), probably *Scoliopleura*, and *Muelleria* ([39]), and their restriction to these genera suggests they comprise a synapomorphy for a group at family or subordinal level. *Muelleria* was not considered by Round et al. (1990, [1]) but shares several characteristics of valve structure with *Neidium* (two-layered valves with striae of small round pores in each layer; presence of a longitudinal canal within the thickness of the valve; development in many species of a flap over the external polar raphe endings, making these appear forked; central internal raphe endings accompanied by a double helictoglossa, though this is elongate in *Muelleria* and almost forms two separate helictoglossae) and Spaulding and Stoermer ([41]) therefore tentatively placed the genus in the Neidiaceae (see also Van de Vijver et al. 2010 [42] for descriptions of *Muelleria* valve morphology). The *Neidium–Biremis–Scoliopleura–Muelleria* group also exhibits similarities with respect to sexual reproduction and auxosporulation, in addition to the possession of incunabular caps discussed above. These similarities include rounding off and rearrangement of the gametes before plasmogamy (e.g. **Fig. 7C, D**); formation of the zygotes wholly within the gametangia; expansion of the auxospores parallel to the apical axes of the gametangia; linear auxospores; a narrow primary transverse perizonial band; and, in allogamous species, trans behavioural anisogamy ([34], [43], [36], [40]), allowing the auxosporulation pattern to be classified as type IA1a in Geitler's scheme ([44]). However, several of these features are widespread among biraphid diatoms. For example, type IA1a allogamous auxosporulation is also found in *Frustulia*, *Amphipleura*, *Gomphonema*, *Cymbella*, *Placoneis* and *Nitzschia* ([44], [45]). So, although the wider aspects of auxosporulation are consistent with the relationships suggested by the concatenated gene tree, they do not give it strong support.

Recently two further diatom genera showing a certain degree of similarity with *Biremis* have been established. These are *Labellicula* Van de Vijver & Lange-Bertalot in Van de Vijver et al. (2005) ([46]) and *Olifantiella* Riaux-Gobin & Compére 2009 ([47]). Whereas *Labellicula* is a monotypic genus with one species described from Subantarctic ([46]), *Olifantiella* seems to be very diverse, especially in the tropical regions and coral reef habitats in particular ([48]). Interestingly, although *Labellicula* and *Olifantiella* bear stigmata (not found in any of the Scoliotropidaceae and Neidiacaeae genera mentioned above), they nevertheless share some characters with *Neidium* (*Labellicula*, cf. [46]) and *Biremis* (*Olifantiella*, cf. [47] and [48]). The similarity between *Labellicula* and *Neidium* lies in the valve internal raphe structure, with double helictoglossae, whereas with *Biremis*, *Labellicula* shares aspects of girdle structure, composed of numerous punctate copulae. *Olifantiella* also has a girdle composed of numerous punctate copulae and double helictoglossae internally, but more interesting is that the chambered transapical striae resemble very much those of *Biremis*. However, since both *Labellicula* and *Olifantiella* show the presence of stigmata, which are missing in *Biremis* and Neidiaceae, further analysis is necessary before any formal conclusion is made about the relationships between them.

## Supporting Information

Appendix S1 **Alignment of concatenated DNA sequence data in NEXUS format used for phylogenetic analyses in this study.** Sequence data for the 3 genes of each taxon start with nuclear-encoded ribosomal small-subunit (bases 1–1764), chloroplast-encoded *rbc*L (bases 1765–3237) and chloroplast-encoded *psb*C (bases 3238–4396). Inserted nucleotides unique to *Biremis panamae* nuclear-encoded ribosomal small-subunit have been removed from this alignment.
(NEX)

Appendix S2 **Alignment of concatenated DNA sequence data in NEXUS format including the inserted nucleotides unique to** ***Biremis panamae*** **nuclear-encoded ribosomal small-subunit.** Sequence data for the 3 genes of each taxon start with nuclear-encoded ribosomal small-subunit (bases 1–3193), chloroplast-encoded *rbc*L (bases 3194–4666) and chloroplast-encoded *psb*C (bases 4667–5825).
(NEX)

Appendix S3 **Maximum likelihood phylogeny (with bootstrap values at nodes) inferred from an alignment of** ***rbc*** **L marker.** *Neidium* sp. NEI323TM, *Neidium* sp. NEI 44, *Neidium* sp. NEI428T and *Neidium* sp. NEI Balk482 represent previously unpublished *rbc*L gene sequences from different *Neidium* species. *Biremis* sp. represents a *rbc*L gene sequence from an unpublished *Biremis* sp. The tree is rooted with the pennate araphid taxa *Ctenophora pulchella* and *Tabularia* cf. *tabulata*. Support values lower than 50% were not included in the tree. The GenBank *Achnanthidium coarctatum* name has been changed to *Achnanthes coarctata*.
(TIF)
