# Supplementary figures and images for: A Description of Biremis panamae sp. nov., a New Diatom Species from the Marine Littoral, with an Account of the Phylogenetic Position of Biremis D.G. Mann et E.J. Cox (Bacillariophyceae)
Source: PLoS One. 2014 Dec 10;9(12):e114508. doi: 10.1371/journal.pone.0114508 (PMC4262420; doi:10.1371/journal.pone.0114508)

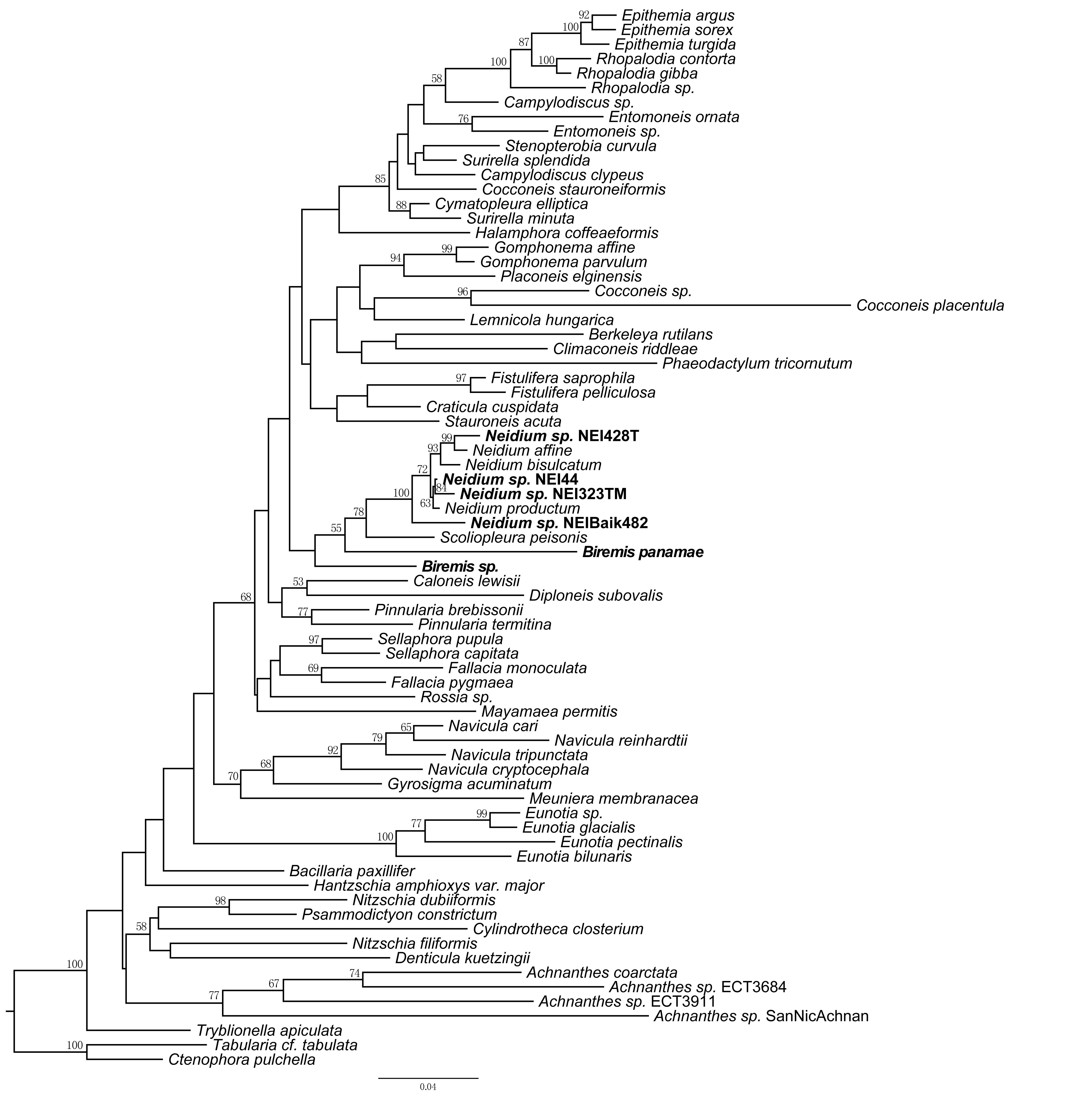

Supplement: Appendix S3 — Maximum likelihood phylogeny (with bootstrap values at nodes) inferred from an alignment of rbc L marker. Neidium sp. NEI323TM, Neidium sp. NEI 44, Neidium sp. NEI428T and Neidium sp. NEI Balk482 represent previously unpublished rbcL gene sequences from different Neidium species. Biremis sp. represents a rbcL gene sequence from an unpublished Biremis sp. The tree is rooted with the pennate araphid taxa Ctenophora pulchella and Tabularia cf. tabulata. Support values lower than 50% were not included in the tree. The GenBank Achnanthidium coarctatum name has been changed to Achnanthes coarctata. (TIF) [file pone.0114508.s003.tif]
